# G4RNA: an RNA G-quadruplex database

**DOI:** 10.1093/database/bav059

**Published:** 2015-06-16

**Authors:** Jean-Michel Garant, Mikael J. Luce, Michelle S. Scott, Jean-Pierre Perreault

**Affiliations:** RNA Group/Groupe ARN, Département de biochimie, Faculté de médecine et des sciences de la santé, Pavillon de recherche appliquée sur le cancer, Université de Sherbrooke, QC J1E 4K8, Canada

## Abstract

G-quadruplexes (G4) are tetrahelical structures formed from planar arrangement of guanines in nucleic acids. A simple, regular motif was originally proposed to describe G4-forming sequences. More recently, however, formation of G4 was discovered to depend, at least in part, on the contextual backdrop of neighboring sequences. Prediction of G4 folding is thus becoming more challenging as G4 outlier structures, not described by the originally proposed motif, are increasingly reported. Recent observations thus call for a comprehensive tool, capable of consolidating the expanding information on tested G4s, in order to conduct systematic comparative analyses of G4-promoting sequences. The G4RNA Database we propose was designed to help meet the need for easily-retrievable data on known RNA G4s. A user-friendly, flexible query system allows for data retrieval on experimentally tested sequences, from many separate genes, to assess G4-folding potential. Query output sorts data according to sequence position, G4 likelihood, experimental outcomes and associated bibliographical references. G4RNA also provides an ideal foundation to collect and store additional sequence and experimental data, considering the growing interest G4s currently generate.

**Database URL:** scottgroup.med.usherbrooke.ca/G4RNA

## Introduction

**Figure 1.**
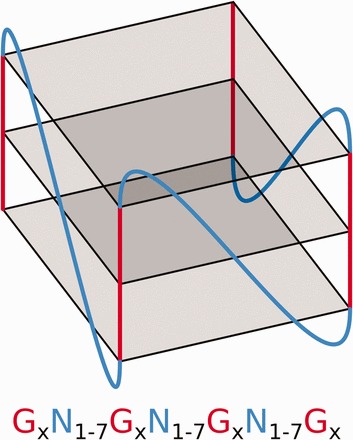
Schematic of an RNA G4 structure and the regular expression used to predict this motif where *N* refers to any base including guanine and × ≥3.

G-quadruplexes (G4s) are tetrahelical structures adopted by guanine-rich nucleic acids. Folding into a G-quartet relies on the planar interaction of four guanines, through Hoogsteen hydrogen bonds ( 1 ). G-quartet formation depends on oxygen-atom charge compensation via recruitment of a monovalent cation to its center, usually a potassium or sodium ion. Stacking of several G-quartets constructs a core G4 structure, the four helical edges of which feature sequential phosphodiester-bonded guanine residues known as ‘G tracks’. The four G tracks at the G4 core are linked up to one another by three separate loops of random nucleotidic composition ( Figure 1 ). G4s are thermodynamically favorable and RNA G4s are generally much stabler than their DNA counterparts, exhibiting relatively higher denaturation temperatures. Structural stability depends on a variety of internal and external features including the presence and number of G-quartets, loop length, occurrence of bulges within G tracks, as well as cation availability and concentration ( 2 , 3 ). Folding probability greatly depends on relative stability and the presence of consecutive cytosine residues upstream and/or downstream of the G4 core. Cytosine-rich sequences tend to favor Watson Crick structures over that of G4s ( 4 ).

The rapidly growing interest in RNA G4s stems from relatively recent reports of their gene-expression regulatory activities. These are mediated through a variety of mechanisms including translational regulation, splicing, polyadenylation and mRNA localization ( 5–8 ). The wide array of G4 functions and their ability to coordinate gene regulation at multiple, post-transcriptional levels are currently driving research in potential therapeutics ( 9 , 10 ), molecular binding ( 11 , 12 ) and the development of molecular tools ( 13 , 14 ). Prediction of G4 folding, which is required for G4 biological activity, is one such area that faces an important challenge. The widely accepted motif used to predict a potential G4 sequence was first described by Huppert and Balasubramanian ( 15 ) nearly a decade ago ( Figure 1 ). Since then, leading experts in the field have never ceased to rethink the definition of a potential G4 sequence ( 16 , 17 ). Comparing a sequence of interest with that of known G4 structures in order to find a close relative, indicative of some likelihood of G4 formation, now requires screening extensive sequence data as well as cross-referencing these against vast amounts of experimental data. G4RNA is a reference database housing human RNA sequences already tested for G4 folding, along with their associated experimental data, provenance and relevant predictive measures. The creation of this comprehensive dataset is one key step toward centralizing invaluable research information and providing a reliable and expert reference tool for conducting systematic comparative sequence analyses.

## Implementation

### Construction of G4RNA

The G4RNA dataset is stored in a relational database built in MySQL (5.5.40). The dataset core consists of nucleotide sequences as well as their associated primary attributes such as sequence identifier in the reference publication, length and position on the hg38 reference genome assembly, and reference gene. G4RNA was entirely populated by manual curation of the literature considering only peer-reviewed publications, and experimentally validated sequences. The nature of experiments performed for each sequence, results confirming or infirming a G4 structure, as well as the original bibliographical references are made available for in-depth investigation.

Output values have also been collated for all G4-promoting sequences using available G4-predictive tools. These values are highly useful for estimating the likelihood of G4 folding. Centralizing such estimates for all available experimentally tested sequences provides a strong basis for powerful systematic comparative analyses. We expect such analyses will accelerate the generation of new insights in the field of RNA biology. Predictive values include RNAfold secondary-structure prediction (RNAfold 2.1.7) ( 18 ), consecutive-guanine over consecutive-cytosine ratio (cGcC score) ( 4 ) and best scored G4 potential using QGRS mapper ( 19 ).

### Web interface

G4RNA is accessible through a web-based browsing tool at the following URL: http://scottgroup.med.usherbrooke.ca/G4RNA/ . A specifically designed query form helps limit output to user-relevant information. Queries can spark two separate search engines, either coordinately or independently, that will browse through the G4RNA dataset. Using key words, the first search engine uncovers matches in the user-chosen attribute of interest. Key word search terms can be stated using a regular expression and the IUPAC nucleotide ambiguity code. The second engine sifts through genomic positions using hg38 annotations.

**Figure 2.**
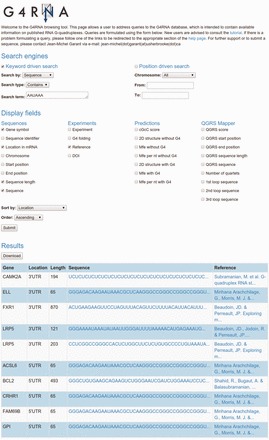
Cropped screen capture of a G4RNA query. It displays the gene symbol, location in the mRNA, nucleotide length, sequence and reference of wild-type sequences presenting an ‘AAUAAA’ polyadenylation signal sorted by their location in mRNA.

Query output is displayed as an HTML-table, with a specific row for every match uncovered and a set of columns each featuring a customizable field as instructed by the user in the query form. Figure 2 depicts an example of a query of wild-type G4RNA sequences containing a “AAUAAA’' polyadenylation signal. Sorting the output by location displays four sequences from 3'UTRs which present a potential regulation of polyadenylation via the folding of a G4 since those sequences contain both a G4 and the signal. Supporting this hypothesis, LRP5 was shown to regulate polyadenylation through the folding of a G4 ( 8 ). The output table can be downloaded in a spreadsheet file format (.xls). The process requires no authentication. The website is a Django (1.6.5) realization, running on an Apache (2.2.22) webserver with an Ubuntu (12.04.4) operating system.

## Results and discussion

The entire G4RNA dataset comprises a total of 334 RNA sequences from 94 separate genes, including 165 distinct wild-type sequences. All dataset sequences have been experimentally tested for secondary-structure G4 folding, with results published in peer-reviewed journals. The dataset collates information from hundreds of experiments. Every sequence has been attributed a specific Boolean value describing whether a given experiment, referenced to the relevant publication, confirmed or infirmed G4 folding. Overall, results from 352 experiments support G4 folding for 185 sequences, whereas 223 experiments support alternate folding structures for 140 other sequences ( Table 1 ).

**Table 1.** Distribution of sequences in G4RNA database

	Locations		G4 validation ^a^		
			Confirmed G4s	Denied G4s	Inconclusive results
Wild-type sequences	5′UTR	99	79	17	3
	3′UTR	45	41	0	4
	Exonic coding	12	10	2	0
	Intronic	8	8	0	0
	TERRA	1	1	0	0
	Total	165	139	19	7
All sequences	5′UTR	218	108	106	4
	3′UTR	72	45	23	4
	Exonic	21	17	4	0
	Intronic	12	9	2	1
	Artificial	10	5	5	0
	TERRA	1	1	0	0
	Total	334	185	140	9
**Techniques**					
Discrete tests	Probing and SHAPE	167	87	80	0
	Circular dichroism	136	92	44	0
	Expression assay	115	58	54	3
	Melting temperature	104	69	35	0
	NMR	20	19	1	0
	Native gel mobility	16	14	2	0
	Other	20	13	7	0
	Total	578	352	223	3

^a^ G4 validation presents the outcome of experimental tests in three columns: Confirmed G4s / denied G4s / inconclusive results

UTR, Untranslated Region; TERRA, Telomeric Repeat-containing RNA; SHAPE, Selective 2'-Hydroxyl Acylation analyzed by Primer Extension; NMR, Nuclear Magnetic Resonance.

A vast majority, i.e. 87% of the G4RNA dataset sequences are found in the UTRs of naturally occurring mRNAs. This is not assumed to be representative of the natural prevalence of G4 structures since it is biased by experimenters’ research interests. In addition, a small set of artificial sequences of particular interest are also featured in the G4RNA Database. On average, each sequence has been experimentally tested using 1.73 techniques, with most confirmed G4s demonstrated using more than a single method. Inventoried techniques are mainly structural-probing methods (In Line, RNase, DMS probing and SHAPE), circular dichroism, expression assays (Luciferase or other protein-expression assays) and melting-point determination using UV thermal-denaturation protocols ( Table 1 ). Data were extracted from 46 peer-reviewed publications ( Table 2 ) through PubMed searches for ‘RNA G-quadruplex, quadruplex, tetraplex’ and using ReadCube’s personalized recommendations of literature based on previous successful search results. The data are updated periodically on a monthly basis.

**Table 2.** Sources of data in the literature

Journals	Publications	Sequences
NAR	12	160
RNA	5	49
Biochemistry	5	24
Nature Group ^a^	5	15
Journal of Biochemical Chemistry	3	8
Other	16	78
Total	46	334

^a^ Nature group comprises Nature, Nature Structural & Molecular Biology and Nature Chemical Biology journals.

The G4RNA Database is not meant to replace current G4 databases such as GRSDB2 ( 20 ) which provide information about the distribution of predicted G4 forming sequences in the transcriptome. G4RNA Database is rather a user-friendly, flexible, expert reference tool. Its expandable dataset provides a solid foundation for the development and validation of future more advanced tools. Indeed, its validated non-G4 folding sequences are crucial for any systematic comparative investigation. They are either non-conclusive potential G4s, G4-derivatives or G4-mutant sequences. Those non-G4 sequences are usually associated with a similar G4 sequence and were used as its negative controls.

For example, the 5′UTR of the AASDHPPT gene was shown to fold into a G4 structure. Its folding is compromised by a single G to C nucleotide polymorphism. Those two sequences are found in the G4RNA database as is the G/A mutant that was used as negative control for testing ( 7 ). These sequences were tested using circular dichroism spectroscopy, in line probing and luciferase expression assay. This example shows how a G4 structure can be affected by minor changes and provides insights into essential features for potential G4s.

## Conclusion

The era of ‘Big Data’ and modern meta-analysis strategies has ushered in new possibilities for the structured, long-term gathering of scientific data and the creation of new knowledge that will accelerate discoveries in many scientific fields ( 21 ). Implementation of the G4RNA Database described here is a logical by-product of the scientific community’s excitement for G4s. Fast and easy access to data describing known G4 will stimulate current research with impact in areas ranging from therapeutics to molecular tools design ( 9–14 ). Gathering of important experimental data is a painstaking process. We encourage RNA research groups to actively share their work by regularly submitting their experimentally validated G4-folding RNA sequences, as well as their non-folding counterparts, to this new expert reference tool which is open to the entire scientific community.

## Supplementary Material

Supplementary Data
