# Supplementary figures and images for: G4RNA: an RNA G-quadruplex database
Source: Database (Oxford). 2015 Jun 16;2015:bav059. doi: 10.1093/database/bav059 (PMC5630937; doi:10.1093/database/bav059)

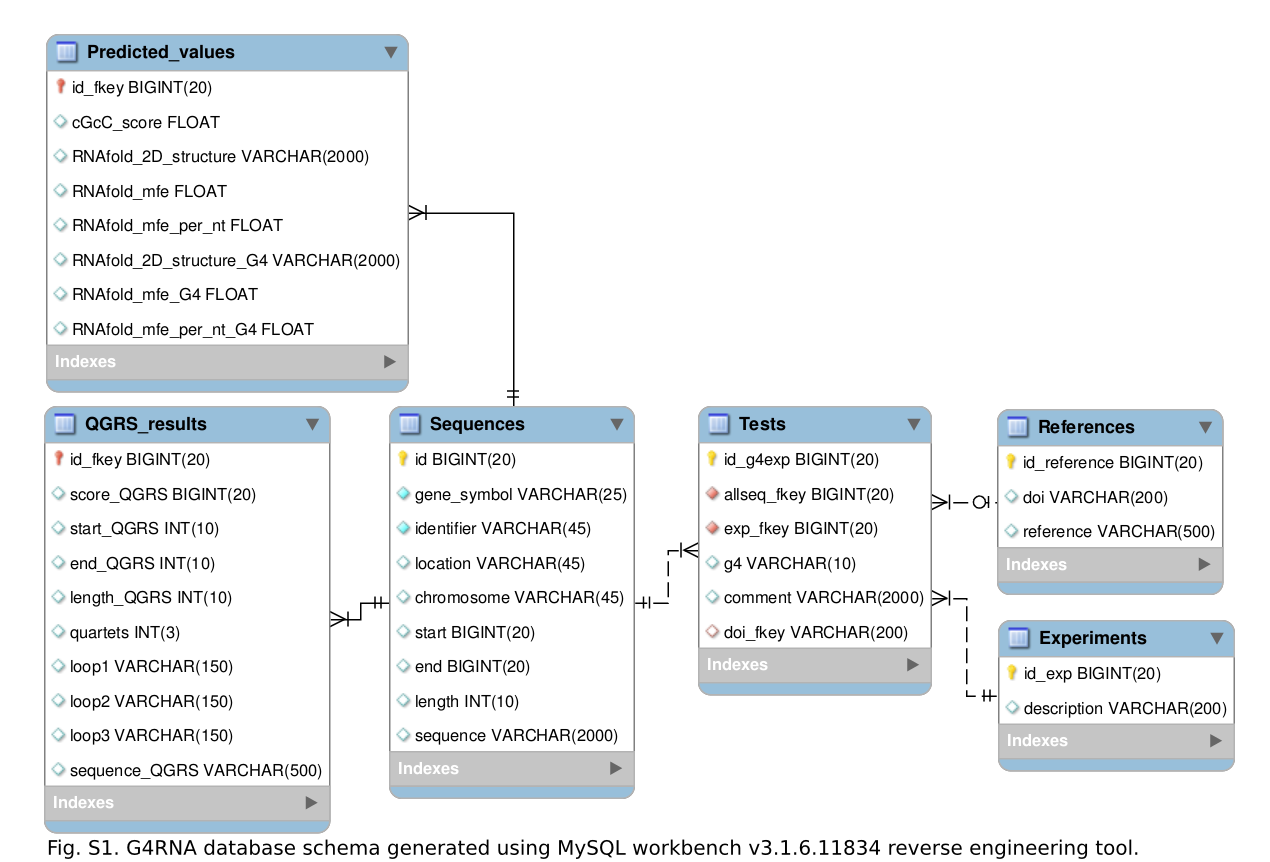

Supplement: Supplementary Data [file bav059_Supplementary_Data.zip › G4RNA_FigS1.png]
